# Supplementary material for: Splice-Junction-Based Mapping of Alternative Isoforms in the Human Proteome
Source: Cell Rep. Author manuscript; Available in PMC 2020 Jan 15. (PMC6961840; doi:10.1016/j.celrep.2019.11.026)
Supplement: 3 [file NIHMS1546469-supplement-3.zip › DF2/PXD009737/TestisTrypsin-362-Q13438-KPPPSPQPTGK.pdf]

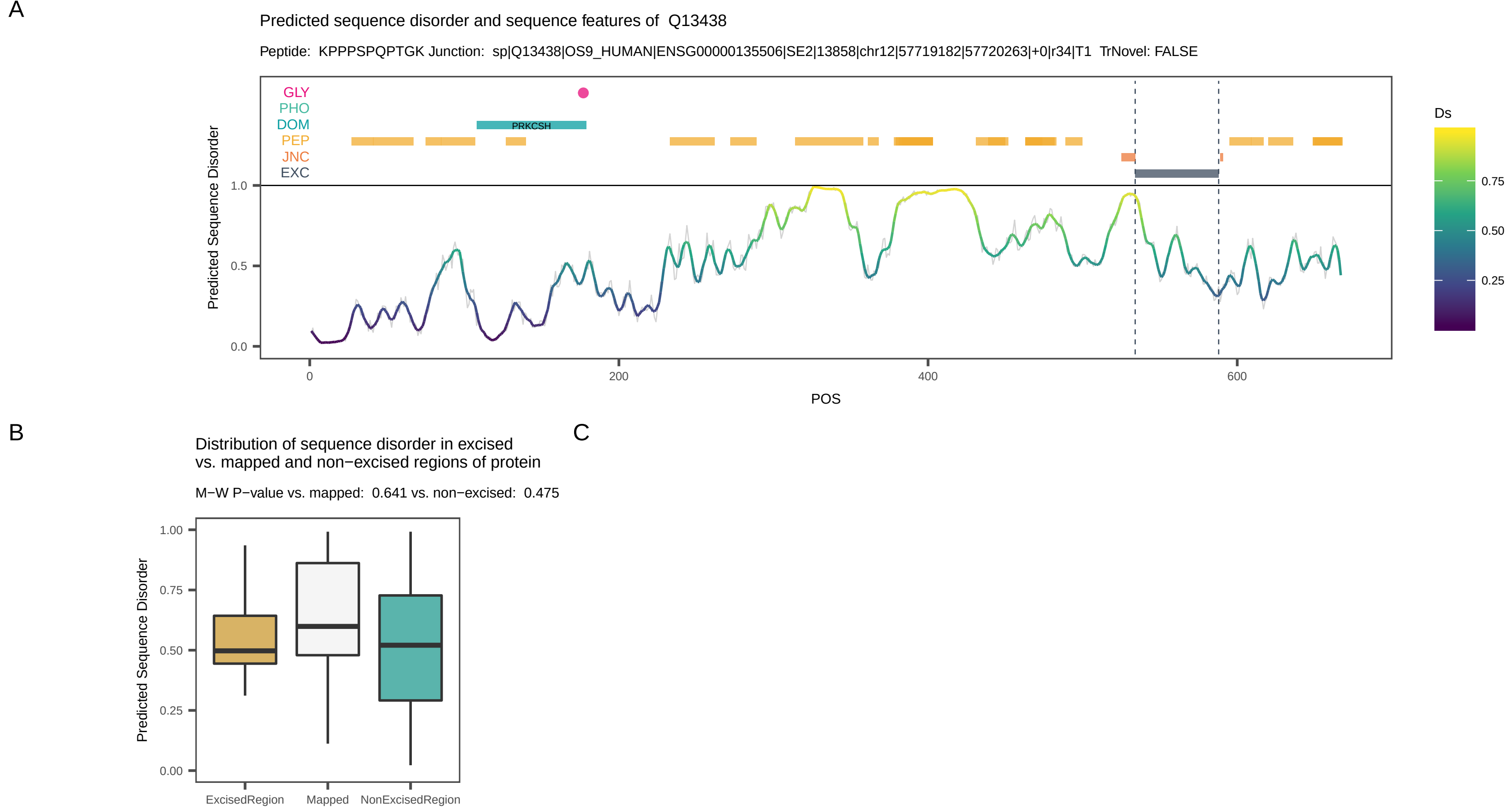
A
Predicted sequence disorder and sequence features of Q13438
Peptide: KPPPSPQPTGK Junction: sp|Q13438|OS9_HUMAN|ENSG00000135506|SE2|13858|chr12|57719182|57720263|+0|r34|T1 TrNovel: FALSE
GLY
PHO
DOM
PEP
JNC
EXC
PRKCSH
Predicted Sequence Disorder
1.0
0.5
0.0
0
200
400
600
POS
Ds
0.75
0.50
0.25
B
Distribution of sequence disorder in excised vs. mapped and non−excised regions of protein
M−W P−value vs. mapped: 0.641 vs. non−excised: 0.475
Predicted Sequence Disorder
1.00
0.75
0.50
0.25
0.00
ExcisedRegion
Mapped
NonExcisedRegion
C
